# Non-TZF Protein AtC3H59/ZFWD3 Is Involved in Seed Germination, Seedling Development, and Seed Development, Interacting with PPPDE Family Protein Desi1 in Arabidopsis

**DOI:** 10.3390/ijms22094738

**Published:** 2021-04-29

**Authors:** Hye-Yeon Seok, Hyungjoon Bae, Taehyoung Kim, Syed Muhammad Muntazir Mehdi, Linh Vu Nguyen, Sun-Young Lee, Yong-Hwan Moon

**Affiliations:** 1 Institute of Systems Biology, Pusan National University, Busan 46241, Korea; seokhyeon@pusan.ac.kr (H.-Y.S.); laptopdog@naver.com (H.B.); 2 Department of Integrated Biological Science, Pusan National University, Busan 46241, Korea; kth3245@naver.com (T.K.); smmms2009@gmail.com (S.M.M.M.); nhqdkr@gmail.com (L.V.N.); 3 Biological Systems and Engineering Division, Lawrence Berkeley National Laboratory, Berkeley, CA 94720, USA; symoonlee@lbl.gov; 4 Department of Molecular Biology, Pusan National University, Busan 46241, Korea

**Keywords:** Arabidopsis, AtC3H59, CCCH zinc finger, non-TZF, PPPDE, seed germination, seed development, seedling development, ZFWD

## Abstract

Despite increasing reports on the function of CCCH zinc finger proteins in plant development and stress response, the functions and molecular aspects of many non-tandem CCCH zinc finger (non-TZF) proteins remain uncharacterized. AtC3H59/ZFWD3 is an Arabidopsis non-TZF protein and belongs to the ZFWD subfamily harboring a CCCH zinc finger motif and a WD40 domain. In this study, we characterized the biological and molecular functions of AtC3H59, which is subcellularly localized in the nucleus. The seeds of *AtC3H59*-overexpressing transgenic plants (OXs) germinated faster than those of wild type (WT), whereas *atc3h59* mutant seeds germinated slower than WT seeds. *AtC3H59* OX seedlings were larger and heavier than WT seedlings, whereas *atc3h59* mutant seedlings were smaller and lighter than WT seedlings. Moreover, *AtC3H59* OX seedlings had longer primary root length than WT seedlings, whereas *atc3h59* mutant seedlings had shorter primary root length than WT seedlings, owing to altered cell division activity in the root meristem. During seed development, *AtC3H59* OXs formed larger and heavier seeds than WT. Using yeast two-hybrid screening, we isolated Desi1, a PPPDE family protein, as an interacting partner of AtC3H59. AtC3H59 and Desi1 interacted via their WD40 domain and C-terminal region, respectively, in the nucleus. Taken together, our results indicate that AtC3H59 has pleiotropic effects on seed germination, seedling development, and seed development, and interacts with Desi1 in the nucleus via its entire WD40 domain. To our knowledge, this is the first report to describe the biological functions of the ZFWD protein and Desi1 in Arabidopsis.

## 1. Introduction

Zinc finger proteins belong to a large family of proteins and are the most abundant proteins in eukaryotic genomes. Zinc finger proteins can be classified into different types according to the order and number of Cys (C) and His (H) residues bound to zinc ions inside the motif [1,2]. CCCH zinc finger proteins contain one or more CCCH zinc finger motif(s) characterized by three Cys residues and one subsequent His residue, and they have been widely found in organisms ranging from bacteria to higher eukaryotes [3]. The specificity of the molecular functions of each zinc finger protein is connected to individual finger and spacing structures [4].

Sixty-eight CCCH zinc finger protein genes classified into 11 subfamilies have been identified in Arabidopsis (*Arabidopsis thaliana*) [3]. CCCH zinc finger proteins are divided into tandem CCCH zinc finger (TZF) and non-TZF proteins. TZF proteins contain two tandem CCCH zinc finger motifs, whereas non-TZF proteins have fewer or greater than two CCCH zinc finger motifs. There are 26 putative TZF proteins and 42 non-TZF proteins in the Arabidopsis genome [5].

TZF genes are required in multiple biological processes such as the regulation of plant growth, maintenance of homeostasis, acquisition of immunity against pathogens, and adaptation to hormone and stress responses in several plant species. In Arabidopsis, overexpression of *AtTZF2*/*AtOZF1* and *AtTZF3*/*AtOZF2* confers ABA hypersensitivity and drought tolerance [6]. Moreover, *AtTZF6*/*PEI1* is involved in embryo formation [7]. *AtTZF14* and *AtTZF15*/*AtCDM1* are involved in secondary wall thickening and anther development [8]. In rice (*Oryza sativa*), expression of *OsTZF1* is upregulated by drought, salt stress, and hydrogen peroxide [9]; *OsC3H10* is involved in drought tolerance by modulating the expression of stress-related genes [10].

However, unlike TZF, the functional roles of non-TZF genes in plants are poorly understood. One of the non-TZF genes in Arabidopsis, *AtC3H37*/*HUA1*, was reported to facilitate *AGAMOUS* pre-mRNA processing causing floral morphogenesis [11]. *AtC3H17* has pleiotropic effects on development as well as in the salt stress response through the regulation of downstream genes [5,12]. Two non-TZF genes in *Brassica campestris*, *BcMF30a* and *BcMF30c*, are involved in pollen development. Appropriate expression levels of these two genes are critical for maintaining normal pollen development [13].

CCCH zinc finger proteins are RNA-binding proteins involved in post-transcriptional regulation. In Arabidopsis, the TZF motif in AtTZF1 is important for the binding of RNA in a zinc-dependent manner [2]. Moreover, AtC3H3 binds to RNA and acts as a ribonuclease [14]. However, plant CCCH zinc finger proteins are also involved in transcriptional regulation. AtC3H14 and AtC3H15/AtCDM1 regulate transcription through DNA-binding and exhibit transactivation activity in yeast [8]. To date, AtC3H17, IbC3H18, PvC3H72, and PdC3H17 have also been reported as transcriptional activators [5,15,16,17].

The WD40 domain was first identified in the β-subunit of the heterotrimeric GTP-binding protein and in the CDC4 protein as repetitive sequence motifs up to 43 amino acids (aa) in length. Each repeat has a highly conserved Gly-His (GH) dipeptide at the N-terminus and a conserved Asp (D) ending with Trp-Asp (WD) dipeptide at the C-terminus [18]. This finding was further redefined as a 44–60 aa motif, where the GH dipeptide is located at residues 11 to 24 from the N-terminus and the WD dipeptide is positioned at the C-terminus [19,20]. WD40 domains with seven WD40 repeats were the most common type found in the identified WD40 proteins [21]. WD40 domains often function as scaffolds for protein-protein interactions to form protein complexes [19,21,22]. Protein complexes, including WD40 domains, play important roles in a wide range of fundamental biological processes and cellular functions such as cell division, transcriptional regulation, DNA damage repair, histone modification, RNA processing, vesicle formation regulation, and vesicular trafficking [23,24,25,26,27,28,29,30].

In Arabidopsis, a new subfamily of WD40 proteins has been defined as ZFWD proteins that contain CCCH zinc finger motif(s) at the N-terminal region [31]. Four ZFWD proteins—ZFWD1, ZFWD2, ZFWD3, and ZFWD4—have been identified and are also known as AtC3H48, AtC3H63, AtC3H59, and AtC3H62, respectively. ZFWD proteins belonging to the CCCH zinc finger protein subfamily IV contain the WD40 domain, which consists of seven WD40 repeats following the CCCH zinc finger motif(s) [3,31]. Although the new subfamily of WD40 proteins and CCCH zinc finger proteins has been defined, the molecular function and biological function of this subfamily remain unknown.

The Permuted Papain fold Peptidases of DsRNA viruses and the Eukaryotes (PPPDE) family has been identified as putative deubiquitinating isopeptidases and is involved in deubiquitination and/or deSUMOylation in mammals [32,33]. Previously, deSUMOylating isopeptidase-1 (DeSI-1) was reported to have deSUMOylating activity in humans [32,34]. In addition, DeSI-2 has a deubiquitinating activity [33]. Previous studies have indicated that DeSI-2 is involved in embryogenesis, apoptosis induction, and cell cycle regulation [35]. However, the function of PPPDE family proteins in plants has not yet been reported, except for that of the Desi3A in Arabidopsis [36].

In this study, we characterized the biological and molecular functions of ZFWD3/AtC3H59 (AtC3H59). ZFWD3/AtC3H59 is involved in Arabidopsis development during germination, seedling development, and seed development. Although ZFWD3/AtC3H59 was subcellularly localized in the nucleus, this did not show transactivation activity. ZFWD3/AtC3H59 interacted with Desi1 via the WD40 domain in the nucleus. Desi1 is known to have a deSUMOylating activity, and ZFWD3/AtC3H59 contains predicted SUMOylation targeted sites within the WD40 domain, implying that ZFWD3/AtC3H59 may be a substrate of Desi1 in regulating Arabidopsis development.

## 2. Results

### 2.1. AtC3H59 Has One CCCH Zinc Finger Domain and One WD40 Domain

**Figure 1:**
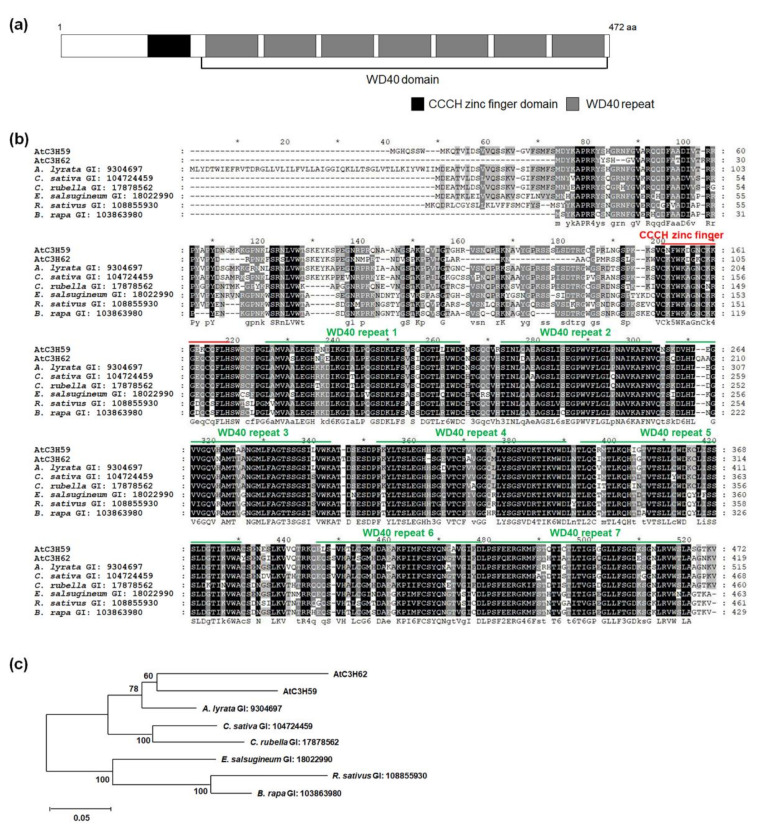
Protein domains of AtC3H59 and conservation of CCCH zinc finger domain and WD40 domain among its paralog and orthologs. (**a**) A CCCH zinc finger domain and a WD40 domain with seven WD40 repeats of AtC3H59 are presented. (**b**) Multiple sequence alignment was carried out with amino acid sequences of full-length ORF of AtC3H59 and its paralog and orthologs using the Clustal Omega program. One conserved CCCH zinc finger domain and WD40 domain region with seven WD40 repeats are annotated. (**c**) Phylogenetic tree of AtC3H59 and its paralog and orthologs was generated with the full-length ORF using maximum likelihood method in MEGA 7.0.26 software. The number on each node indicates the bootstrap value for 1000 replicates. In (**b**,**c**), the GI number of each protein sequence is as follows: AtC3H59, 834089; AtC3H62, 834979; *Arabidopsis lyrata*, 9304697; *Camelina sativa*, 104724459; *Capsella rubella*, 17878562; *Eutrema salsugineum*, 18022990; *Raphanus sativus*, 108855930; *Brassica rapa*, 103863980.

AtC3H59 was selected to functionally characterize non-TZF genes in Arabidopsis. AtC3H59 is a member of the ZFWD subfamily that has both CCCH zinc finger motif(s) and a WD40 domain. AtC3H59 has one CCCH zinc finger motif, which can be denoted as C-X_8_-C-X_5_-C-X_3_-H and one WD40 domain, containing seven WD40 repeats (Figure 1a). BLASTP analysis identified one paralog (AtC3H62) and several orthologs in *Arabidopsis lyrata*, *Camelina sativa*, *Capsella rubella*, *Eutrema salsugineum*, *Raphanus sativus*, and *Brassica rapa*. Multiple sequence alignment of AtC3H59 and its paralog and orthologs showed that amino acid sequences were highly conserved, especially in the CCCH zinc finger motif and WD40 domain (Figure 1b). We generated a phylogenetic tree to compare the phylogenetic relationships between AtC3H59 and its paralog and orthologs. AtC3H62 was the most closely related protein and the ortholog in *A. lyrata* was the most closely related to AtC3H59 among orthologs (Figure 1c).

### 2.2. Expression of AtC3H59 during Development and in Organs of Arabidopsis

**Figure 2:**
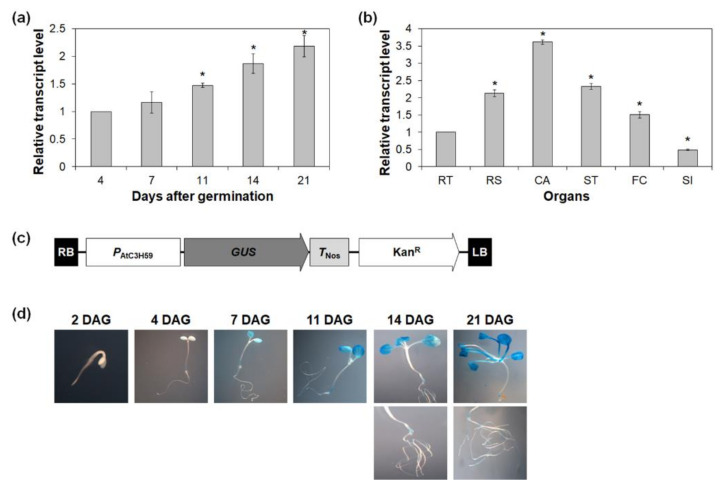
Temporal and spatial expression patterns of *AtC3H59*. (**a**) Quantitative RT-PCR analysis of *AtC3H59* in 4-, 7-, 11-, 14-, and 21-day-old WT seedlings grown under SD conditions. *GAPc* was used for an internal control. Transcript level at 4 DAG was set as 1. (**b**) Quantitative RT-PCR analysis of *AtC3H59* expression in organs of 49-day-old WT grown under LD conditions. *GAPc* was used for an internal control. Transcript level in RT was set as 1. RT, roots; RS, rosette leaves; CA, cauline leaves; ST, stems; FC, floral clusters; SI, siliques. (**c**) Schematic maps of *P*_AtC3H59_::*GUS* for GUS assays. (**d**) Histochemical assay of GUS expression in T_2_ transgenic Arabidopsis plants carrying *P*_AtC3H59_::*GUS* at different developmental stages grown under SD conditions. Representative GUS staining results are shown here. In (**a**,**b**), three independent reactions were performed for each technical replicate. Two technical replicates were performed for each biological replicate. Data shown are the mean ± S.D. (*n* = 6). At least two biological replicates showed similar results, with one shown here. * indicate *t*-test *p* < 0.05.

To gain insight into the potential functions of *AtC3H59*, spatial and temporal expression patterns of *AtC3H59* were examined in various developmental stages and plant organs by quantitative RT-PCR. The transcript level of *AtC3H59* increased as the plants developed from 4 to 21 days after germination (DAG) (Figure 2a). In mature Arabidopsis plants, *AtC3H59* was highly expressed in cauline leaves compared to other organs examined, such as roots, rosette leaves, stems, floral clusters, and siliques (Figure 2b). Semi-quantitative RT-PCR analysis also showed results similar to those obtained with quantitative RT-PCR (Figure S1).

To visualize the spatial and temporal expression patterns of *AtC3H59*, *P*_AtC3H59_::*GUS* transgenic plants were generated and analyzed. The 242 bp upstream region from the transcriptional start site was fused to *GUS* (Figure 2c). Expression of GUS in 7-, 11-, 14-, and 21-day-old seedlings was detected in the cotyledons, root junctions, and roots, and the expression pattern increased as the plants developed (Figure 2d), indicating that the promoter activity of *AtC3H59* increased as plants grew during seedling development. This result is consistent with the expression pattern of *AtC3H59* obtained by quantitative RT-PCR (Figure 2a).

### 2.3. AtC3H59 Protein Is Subcellularly Localized in the Nucleus and Does Not Have Transactivation Activity

**Figure 3:**
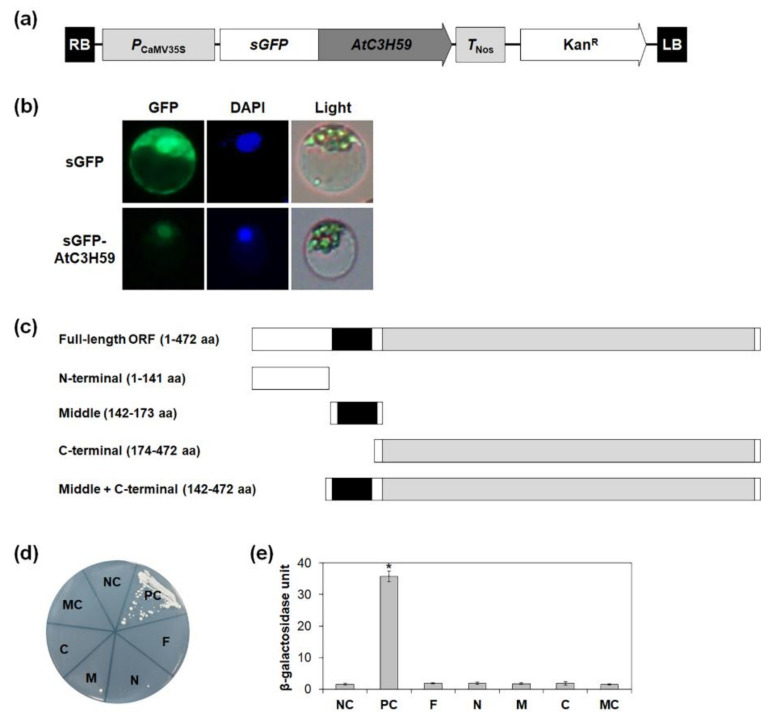
Subcellular localization and transactivation activity of AtC3H59. (**a**) Schematic map of sGFP-fused full-length AtC3H59 construct. (**b**) Subcellular localization of AtC3H59 was examined by transient expression of sGFP-AtC3H59 fusion proteins in Arabidopsis protoplasts. Left, GFP signal; middle, 4′,6-diamidino-2-phenylindole (DAPI) staining; right, light microscopic picture. (**c**) Schematic maps of full-length ORF of AtC3H59 and truncated fragments of AtC3H59 for analysis of transactivation activity in yeast. (**d**) Yeast growth assay. Yeast transformants were grown on SM-Trp/-Ura. (**e**) Quantitative β-galactosidase ONPG assay. The transactivation activities were quantified by measuring the β-galactosidase activity in yeast extract. The data shown are the means ± S.D. (*n* = 3). * *t*-test *p* < 0.05. In (**d**,**e**), pBD-GAL4 vector itself was used as a negative control. NC, negative control; PC, positive control; F, full-length ORF; N, N-terminal region; M, middle region; C, C-terminal region; MC, middle + C-terminal region.

To dissect the molecular actions of *AtC3H59* gene products in plant cells, the subcellular localization of AtC3H59 protein was investigated using the sGFP-fused AtC3H59 construct expressed in Arabidopsis protoplasts (Figure 3a). GFP signals of the sGFP-AtC3H59 construct were observed to be exclusively found in the nucleus, where the GFP signals overlapped with DAPI signals (Figure 3b), suggesting that AtC3H59 has roles in the nucleus.

Non-TZF proteins, such as AtC3H17 and OsLIC, have transactivation activity [5,37]. Therefore, the transactivation activity of AtC3H59 was tested using yeast two-hybrid system. In addition to the full-length open reading frame (ORF) of AtC3H59, its partial fragments were also tested, including the N-terminal region that lacks a domain, the middle region containing the CCCH zinc finger motif, the C-terminal region containing the WD40 domain, and the middle + C-terminal region containing both the CCCH zinc finger motif and the WD40 domain (Figure 3c). Quantitative β-galactosidase 2-nitrophenyl-β-D-galactopyranoside (ONPG) and yeast growth assays showed that neither the full-length AtC3H59 ORF nor any partial fragment of AtC3H59 displays transactivation activity (Figure 3d,e), indicating that AtC3H59 does not have transactivation activity.

### 2.4. AtC3H59 Is Involved in Regulation of Seed Germination and Seedling Development

To analyze the biological functions of *AtC3H59* during Arabidopsis development, we generated *AtC3H59*-overexpressing transgenic plants (OXs) where *AtC3H59* was overexpressed under the control of a modified CaMV 35S promoter and selected three independent T_1_ lines that showed higher expression of *AtC3H59* than WT (Figure S2a–c). In addition, two T-DNA-inserted *atc3h59* mutants, SALK_066026 (*atc3h59-1*) and SALK_045202 (*atc3h59-2*), which contained T-DNA inserted in the first exon of *AtC3H59*, were obtained from the SIGnAL Collection at the Salk Institute (Figure S2d). Homozygous *atc3h59-1* and *atc3h59-2* mutants were selected, and the lack of *AtC3H59* expression in these mutants was confirmed by semi-quantitative RT-PCR (Figure S2e).

**Figure 4:**
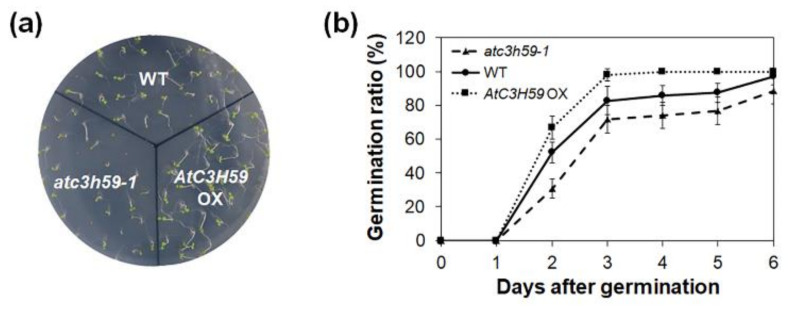
Germination of *AtC3H59* OXs and *atc3h59* mutants. (**a**) Five-day-old WT, *AtC3H59* OXs, and *atc3h59* mutants grown on MS plates under SD condition. (**b**) Germination rate and germination ratio of WT, *AtC3H59* OXs, and *atc3h59* mutants were measured at designated times after sowing on MS plates under SD conditions. Germination was determined based on radicle protrusion. Data shown are the mean ± S.D. (*n* = 30). At least three biological replicates showed similar results. Three independent T_1_ lines of *AtC3H59* OXs showed very similar results, with one shown here.

In the phenotypic analysis, the seed germination rate was first assessed using WT, *AtC3H59* OXs, and *atc3h59* mutants. *AtC3H59* OX seeds germinated faster than WT seeds, whereas *atc3h59* mutant seeds germinated slightly slower than WT seeds (Figure 4). However, the final germination ratio was not altered in *AtC3H59* OXs or *atc3h59* mutants compared with that in WT (Figure 4), indicating that *AtC3H59* is involved in the germination rate.

**Figure 5:**
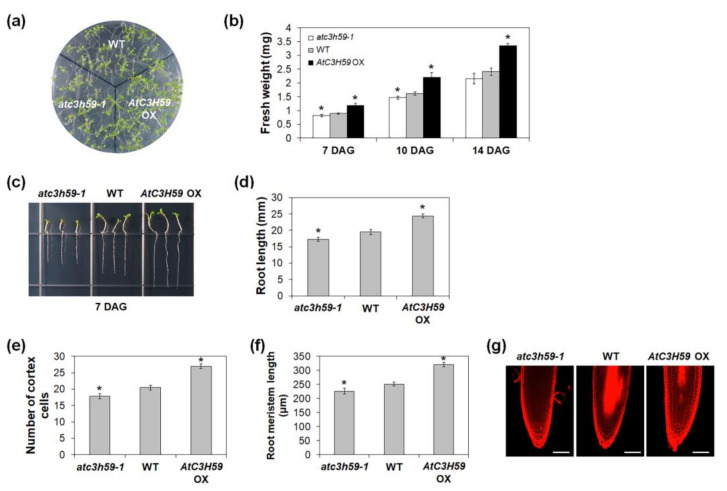
Seedling development of *AtC3H59* OXs and *atc3h59* mutants. (**a**) Fourteen-day-old WT, *AtC3H59* OXs, and *atc3h59* mutants grown on MS plates under SD conditions. (**b**) Fresh weights of shoots of WT, *AtC3H59* OX, and *atc3h59* mutant seedlings grown on MS plates under SD conditions at 7, 10, and 14 DAG. Data shown are the mean ± S.D. (*n* = 5). (**c**) Elongation of primary roots of WT, *AtC3H59* OXs, and *atc3h59* mutants at 7 DAG. (**d**) Primary root lengths of WT, *AtC3H59* OXs, and *atc3h59* mutants grown on MS plates under SD conditions were measured at 7 DAG. Data shown are the mean ± S.D. (*n* = 24). (**e**) The number of root meristem cortex cells of WT, *AtC3H59* OXs, and *atc3h59* mutants grown on MS plates under SD conditions were measured at 7 DAG. Data shown are the mean ± S.D. (*n* = 12). (**f**) Root meristem length of WT, *AtC3H59* OXs, and *atc3h59* mutants grown on MS plates under SD conditions were measured at 7 DAG. Data shown are the mean ± S.D. (*n* = 12). (**g**) Confocal microscopy of roots of WT, *AtC3H59* OXs, and *atc3h59* mutants grown on MS plates under SD conditions at 7 DAG. Roots were excised from seedlings, stained with 10 μM propidium iodide, and examined by confocal microscopy. Scale bars represent 50 μm. In (**b**,**d**–**f**), * indicate *t*-test *p* < 0.05. At least three biological replicates showed similar results. Three independent T_1_ lines of *AtC3H59* OXs showed very similar results, with one shown here.

During seedling development, the fresh weights of *AtC3H59* OX seedlings were heavier than those of WT seedlings, whereas the fresh weights of *atc3h59* mutant seedlings were lighter than WT seedlings when grown on MS agar plates from 7 to 14 DAG (Figure 5a,b). Moreover, the difference in the fresh weights between *AtC3H59* OXs and WT and between *atc3h59* mutants and WT increased as seedlings developed (Figure 5b). The primary root length of *AtC3H59* OXs was longer than that of WT, whereas the primary root length of *atc3h59* mutants was shorter than that of WT (Figure 5c,d). To determine the role of *AtC3H59* in root development, we analyzed the root cells of WT, *AtC3H59* OXs, and *atc3h59* mutants. Since cell division activity in roots is reflected by root meristem size [38], we compared the number of root meristem cortex cells, including cells from the quiescent center to the first elongated cell, as well as root meristem size. The number of cortex cells in *AtC3H59* OXs was higher than that in WT, whereas *atc3h59* mutants had lower numbers than WT. The root meristem size of *AtC3H59* OXs was larger than that of WT, whereas the root meristem size of *atc3h59* mutants was smaller than that of WT (Figure 5e–g), indicating that cell division activity in roots is regulated by *AtC3H59*. Our results suggest that *AtC3H59* is involved in the development of both shoots and roots during seedling development.

### 2.5. AtC3H59 Is Also involved in Regulation of Seed Development

**Figure 6:**
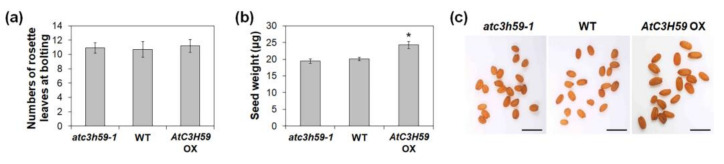
Flowering time and seed development of *AtC3H59* OXs and *atc3h59* mutants. (**a**) The number of rosette leaves of WT, *AtC3H59* OX, and *atc3h59* mutant plants at bolting. Data shown are the mean ± S.D. (*n* = 15). (**b**) Seed weight of WT, *AtC3H59* OXs, and *atc3h59* mutants. Data shown are the mean ± S.D. (*n* = 10). * indicate *t*-test *p* < 0.05. (**c**) Phenotypes of harvested seeds of WT, *AtC3H59* OXs, and *atc3h59* mutants. Scale bars represent 1 mm. At least three biological replicates showed similar results. Three independent T_1_ lines of *AtC3H59* OXs showed very similar results, with one shown here.

Next, we checked whether *AtC3H59* has a function in reproductive development of Arabidopsis. To this end, we analyzed the phenotype of *AtC3H59* OXs and *atc3h59* mutants at the mature stage. First, *AtC3H59* OXs and *atc3h59* mutants did not show significant differences in flowering time compared with WT (Figure 6a). Notably, *AtC3H59* OXs exhibited differences in seed development. The size and weight of *AtC3H59* OX seeds were larger and heavier, respectively, than those of WT seeds (Figure 6b,c), demonstrating that *AtC3H59* is involved in seed development. In contrast, there were no phenotypic differences between *atc3h59* mutants and WT observed during seed development (Figure 6b,c).

### 2.6. AtC3H59 Interacts with Desi1 in the Arabidopsis Nucleus

The WD40 domain is involved in protein-protein interactions [21]. To identify the interacting partner(s) of AtC3H59, we performed yeast two-hybrid screening using a cDNA library of Arabidopsis seedlings. A total of 150 colonies were initially obtained from 5.4 × 10^6^ yeast transformants screened by growth assay using *HIS3* and *ADE2* as reporter genes. Plasmid DNAs with an activation domain (AD) were isolated from the yeast colonies, and we selected 24 positive plasmid DNAs representing seven individual genes (Tables S1 and S2, and Figure S3). Among the seven genes identified, At3g07090, encoding Desi1, belonging to the PPPDE family, was finally selected for further study.

**Figure 7:**
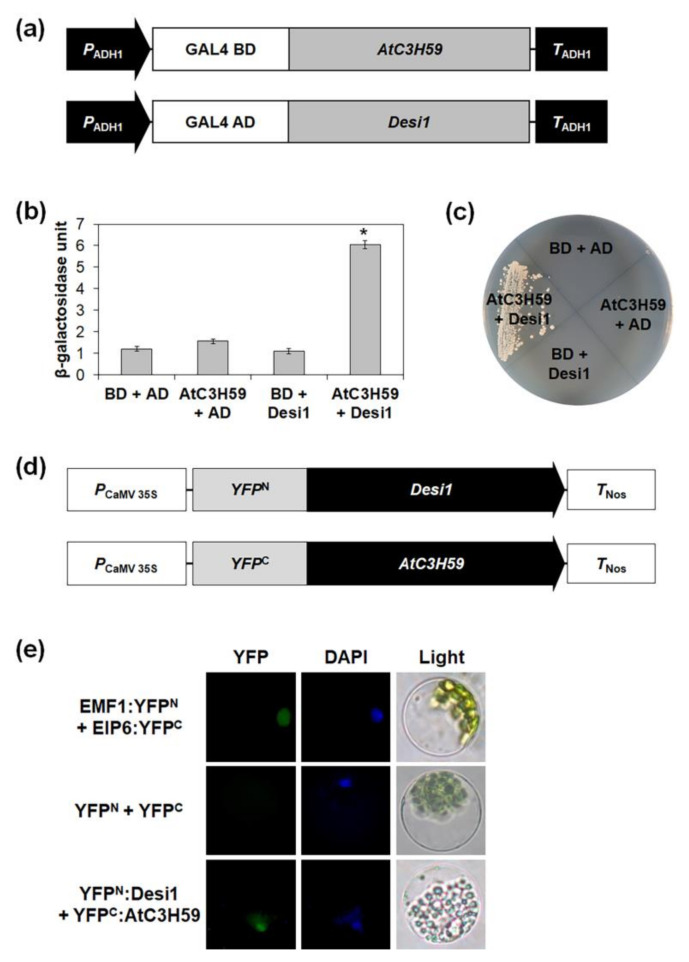
Interaction between AtC3H59 and Desi1. (**a**) Schematic maps of vectors for yeast two-hybrid of AtC3H59 and Desi1. (**b**) Quantitative β-galactosidase ONPG assay. The interaction was quantified by measuring the β-galactosidase activity in yeast extract. Data shown are the means ± S.D. (*n* = 3). * *t*-test *p* < 0.05. (**c**) Yeast growth assay. Yeast transformants were grown on SM-Trp/-Leu/-Ura. (**d**) Schematic maps of vectors for BiFC assay. (**e**) The interaction between AtC3H59 and Desi1 using the BiFC system. The vectors for BiFC assay were transiently expressed in Arabidopsis protoplasts. EMF1 and EIP6 were used as a positive control. Left, YFP signal; middle, 4′,6-diamidino-2-phenylindole (DAPI) staining; right, light microscopic picture. In (**b**,**c**), pBD-GAL4 vector and pGADT7 were used as a bait and a prey for a negative control, respectively.

We validated the interaction between AtC3H59 and Desi1 in yeast using a yeast two-hybrid assay. The GAL4 DNA-binding domain (BD) was fused to the full-length AtC3H59 ORF, and the GAL4 AD was fused to the full-length Desi1 ORF; then, the two constructs were co-transformed into yeast (Figure 7a). The yeast growth and β-galactosidase ONPG assays showed that AtC3H59 strongly interacted with Desi1 (Figure 7b,c and Figure S4a).

Next, to determine whether the interaction between AtC3H59 and Desi1 occurs in Arabidopsis, we investigated the interaction between AtC3H59 and Desi1 in Arabidopsis protoplasts using the BiFC assay. The N-terminal region of YFP fused to the full-length Desi1 ORF and the C-terminal region of YFP fused to the full-length AtC3H59 ORF were co-transformed into Arabidopsis protoplasts (Figure 7d). We observed YFP signals in the nucleus (Figure 7e), suggesting that AtC3H59 interacts with Desi1 in Arabidopsis protoplasts and their interaction occurs in the nucleus.

Previously, it was reported that Desi1 is subcellularly localized in both the nucleus and cytoplasm in tobacco cells [39]. We analyzed the subcellular localization of Desi1 in Arabidopsis cells, and confirmed that this occurred in the nucleus and cytoplasm in Arabidopsis protoplasts (Figure S5). To determine the expression patterns of *Desi1*, the transcript abundance of *Desi1* was examined at various developmental stages using quantitative RT-PCR. *Desi1* was constitutively expressed as the plant developed (Figure S6).

**Figure 8:**
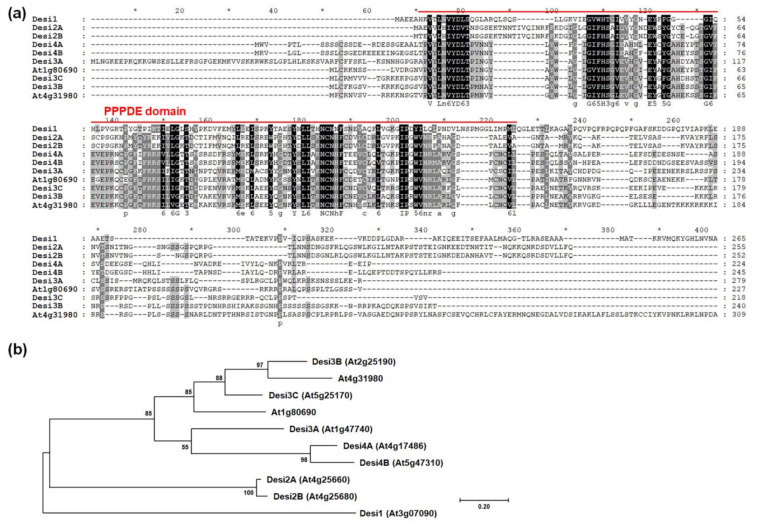
Multiple alignment and phylogenetic tree of PPPDE family proteins in Arabidopsis. (**a**) Multiple sequence alignment was carried out with amino acid sequences of full-length ORF of Arabidopsis PPPDE family proteins using the Clustal Omega program. One conserved PPPDE domain is annotated. (**b**) Phylogenetic tree of Arabidopsis PPPDE family proteins generated with the full-length ORF using maximum likelihood method in MEGA 7.0.26 software. The number on each node indicates the bootstrap value for 1000 replicates.

Database analysis identified 10 proteins from the PPPDE family in Arabidopsis. We performed multiple alignments using amino acid sequences of these proteins to identify any conserved domain(s). Only the amino acid sequences of the PPPDE domain were conserved among the 10 PPPDE proteins (Figure 8a). We also generated a phylogenetic tree to compare the phylogenetic relationships using the full-length amino acid sequences of the 10 Arabidopsis PPPDE proteins. Among the 10 Arabidopsis PPPDE proteins, Desi2A and Desi2B had a close phylogenetic relationship that was distinct from the relationships among seven other PPPDE proteins, namely Desi3A, Desi3B, Desi3C, Desi4A, Desi4B, At4g31980, and At1g80690 (Figure 8b). However, Desi1 did not have a phylogenetic relationship with any of the PPPDE proteins in Arabidopsis (Figure 8b).

### 2.7. AtC3H59 Interacts with Desi1 via the WD40 Domain

**Figure 9:**
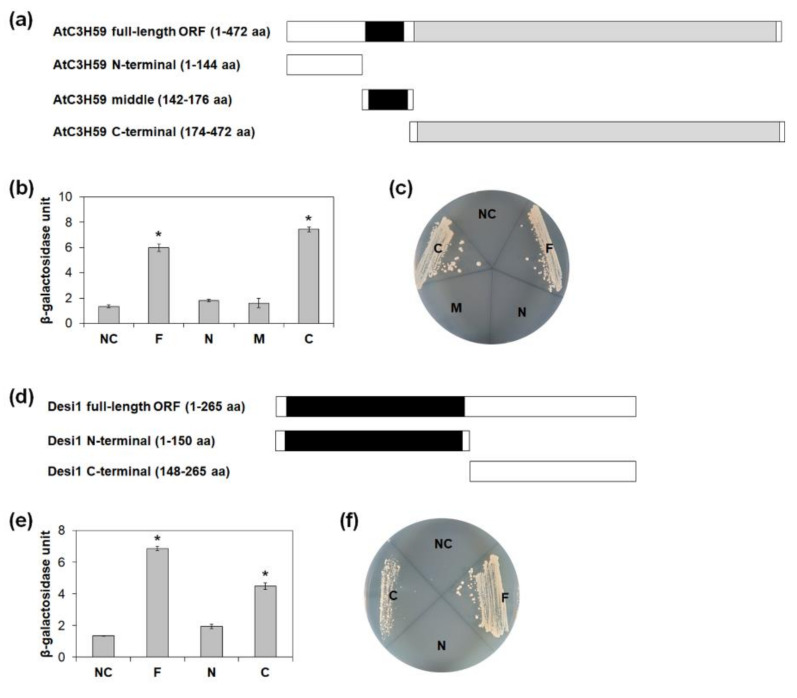
Identification of protein-interacting domains of AtC3H59 and Desi1. (**a**) Schematic maps of full-length ORF of AtC3H59 and truncated fragments of AtC3H59 for yeast two-hybrid with full-length Desi1. (**b**) Quantitative β-galactosidase ONPG assay. The interaction was quantified by measuring the β-galactosidase activity in yeast extract. (**c**) Yeast growth assay. Yeast transformants were grown on SM-Trp/-Leu/-Ura. (**d**) Schematic maps of full-length ORF of Desi1 and truncated fragments of Desi1 for yeast two-hybrid with C-terminal region of AtC3H59. (**e**) Quantitative β-galactosidase ONPG assay. The interaction was quantified by measuring the β-galactosidase activity in yeast extract. (**f**) Yeast growth assay. Yeast transformants were grown on SM-Trp/-Leu/-Ura. In (**b**,**e**), data shown are the means ± S.D. (*n* = 3). * *t*-test *p* < 0.05. In (**b**,**c**,**e**,**f**), pBD-GAL4 vector and pGADT7 were used as a bait and a prey for a negative control, respectively. NC, negative control; F, full-length ORF; N, N-terminal region; M, middle region; C, C-terminal region.

To determine the region of AtC3H59 responsible for the interaction with Desi1, we analyzed the interaction using three GAL4 BD-fused partial fragments of AtC3H59, including the N-terminal, middle region, and C-terminal regions (Figure 9a). Quantitative β-galactosidase ONPG and yeast growth assays showed that the C-terminal region of AtC3H59 containing the WD40 domain interacts with Desi1, whereas the N-terminal region lacking a domain and the middle region containing the CCCH zinc finger motif did not (Figure 9b,c).

The WD40 domain of AtC3H59 consists of seven WD40 repeats (Figure 1a). We tested which WD40 repeat(s) was responsible for the interaction between AtC3H59 and Desi1. To this end, the seven WD40 repeats of AtC3H59 were divided into three regions: WD40-1 containing the first, the second, and the third WD40 repeats; WD40-2 containing the fourth and the fifth WD40 repeats; and WD40-3 containing the sixth and the seventh WD40 repeats (Figure S7a,b). Quantitative β-galactosidase ONPG and yeast growth assays showed that none of the partial repeats of WD40 interacted with Desi1, whereas an interaction occurred when the entire WD40 domain was present (Figure S7c,d), indicating that the entire WD40 domain of AtC3H59 was necessary for the interaction with Desi1.

Desi1 contains a PPPDE domain in the N-terminal region. To identify the domain of Desi1 responsible for the interaction with AtC3H59, we divided Desi1 into two regions; the N-terminal region containing the PPPDE domain and the C-terminal region, and generated GAL4 AD-fusion constructs (Figure 9d). We analyzed the interaction between the partial fragments of Desi1 and AtC3H59 in yeast. In quantitative β-galactosidase ONPG and yeast growth assays, the C-terminal region of Desi1 interacted with AtC3H59, whereas the N-terminal region did not (Figure 9e,f). Our results demonstrated that the entire WD40 domain of AtC3H59 and the C-terminal region of Desi1 are responsible for this interaction. Although the C-terminal region of Desi1 did not have any well-known domain, the amino acid sequences were well conserved among Desi1 and its orthologs (Figure S8), indicating that the conserved sequences might be involved in the protein-protein interactions.

## 3. Discussion

There are 68 CCCH zinc finger genes in Arabidopsis divided into 11 subfamilies [3]. Recently, functional studies on several CCCH zinc finger genes have been performed. CCCH zinc finger genes have been shown to be involved in the regulation of plant growth, maintenance of homeostasis, acquisition of immunity against pathogens, and adaptation to hormone and stress responses. CCCH zinc finger proteins have been identified as RNA-binding proteins and transcriptional regulators. However, the functions of non-TZF genes have been much less reported than those of TZF genes. In this study, we characterized the molecular and biological functions of *AtC3H59*, an Arabidopsis non-TZF gene.

Our phenotype analysis showed that *AtC3H59* is involved in seedling development. *AtC3H59* OX seedlings were larger and heavier than WT seedlings, whereas *atc3h59* mutant seedlings were smaller and lighter than WT seedlings (Figure 5a,b). The difference in fresh weight between *AtC3H59* OXs and WT at 14 DAG was higher than that at 7 and 10 DAG (Figure 5b). Moreover, *AtC3H59* is involved in root development during seedling development via regulation of cell division activity in the root meristem (Figure 5c–f), demonstrating that *AtC3H59* plays an important role in both aerial and root parts during seedling development.

*AtC3H59* OXs displayed larger and heavier seed formation than WT (Figure 6b,c). Previously, we identified the function of the non-TZF gene, *AtC3H17*. *AtC3H17* OXs showed similar phenotypes to *AtC3H59* OXs, including high seed germination rate, large seedling development, and large and heavy seed development via transactivation of seed storage protein genes such as *CRU3*, *OLEO1*, and *OLEO2* [5]. These results suggest that non-TZF proteins, including AtC3H59 and AtC3H17, show pleiotropic effects during development.

AtC3H59 belongs to the ZFWD subfamily and contains a WD40 domain as well as a CCCH zinc finger motif (Figure 1a). There are four ZFWD proteins in Arabidopsis, and all four ZFWD proteins also belong to the CCCH zinc finger protein subfamily IV in Arabidopsis. Among them, two ZFWD proteins, ZFWD1 and ZFWD2, which contain two CCCH zinc finger motifs, are TZFs, and two ZFWD proteins, AtC3H59 and AtC3H62, which contain one CCCH zinc finger motif, are non-TZFs [3,31]. There are homologous ZFWD proteins in other plant species, such as rice, cotton, maize, poplar, pine tree, and ice plant, but there are no animal homologs [31], demonstrating that the ZFWD family is specific to plants. To date, the biological functions of ZFWD proteins have not been reported. This study is the first to demonstrate the biological function of ZFWD proteins.

The WD40 domain is known to be involved in protein-protein interactions [21]. AtC3H59 also interacted with the PPPDE family protein Desi1 via its WD40 domain (Figure 9b,c). In Arabidopsis, JGB contains seven WD40 repeats and interacts with TCP4 to form a regulatory complex that control pollen JA synthesis, ensuring pollination in moist environments [40]. The WD40 domain of AtC3H59 consists of seven WD40 repeats, and the entire WD40 domain, including all seven repeats, was necessary for the interaction between AtC3H59 and Desi1 (Figure 1a and Figure S7). The WD40 repeats in a protein are folded into a β-propeller architecture [21]. In principle, a single WD40 β-propeller can contain four to eight WD40 repeats. So far, only seven- or eight-blade WD40 β-propellers have been structurally confirmed. Interestingly, based on geometry modeling, it was predicted that the seven-fold β-propeller is the most ideal β-sheet geometry. Consistently, seven-fold β-propeller proteins dominated the solved WD40 structures and identified WD40 proteins [21].

Desi1 is a member of the PPPDE family. Members of this family have been identified as a putative deubiquitinating isopeptidases and are involved in deubiquitination and/or deSUMOylation in mammals [32,33,34]. Previous studies have indicated that DeSI-2 is involved in embryogenesis, apoptosis induction, and cell cycle regulation in humans [35]. DeSI-2 deubiquitinates RPS7 and stabilizes this. Several studies have revealed the vital role of RPS7 in modulating the MDM2-p53 pathway in cell proliferation, apoptosis, tumorigenesis, and metastasis [33]. Interestingly, *AtC3H59* is involved in the regulation of cell division in the root meristem (Figure 5c–e). The interaction between Desi1 and AtC3H59 may be related to cell division. Further studies are necessary to reveal this.

Recently, it was reported that Desi3A deSUMOylates FLS2 in Arabidopsis. SUMOylation of FLS2 activates FLS2-mediated immune signaling and deSUMOylation of FLS2 by Desi3A inhibits FLS2 [36]. RACK1B, a WD40 protein, is SUMOylated at its four residues, K50, K276, K281, and K291. SUMOylation increases the stability of RACK1B by blocking ubiquitin conjugation and ubiquitin-mediated degradation [41]. It is possible that Desi1 deSUMOylates its interacting proteins. We analyzed the predicted SUMOylation residue(s) in AtC3H59 using GPS-SUMO [42]. There were three predicted SUMOylation residues, K187, K457, and K471, in AtC3H59 (data not shown), indicating that AtC3H59 might be post-translationally regulated by SUMOylation and/or deSUMOylation by Desi1. Further studies are required to clarify whether AtC3H59 is SUMOylated and/or deSUMOylated by Desi1.

Taken together, our results indicate that AtC3H59 containing a CCCH zinc finger motif and a WD40 domain is a nuclear protein and is involved in seed germination, seedling development, and seed development. AtC3H59 interacts with Desi1 in the nucleus via its WD40 domain. This is the first study to show the biological functions of the ZFWD protein and Desi1 in Arabidopsis. Our study on *AtC3H59* could help expand our understanding of the functions of non-TZF genes.

## 4. Materials and Methods

### 4.1. Plant Materials and Growth Conditions

All *Arabidopsis thaliana* plants used in this study were of the Columbia ecotype. For surface-sterilization, seeds were dipped for 1 min in 70% ethanol, followed by dipping for 10 min in 1/10-diluted commercial bleach (0.4% NaOCl), and then washing with sterile distilled water four times. The seeds were placed in the dark for 2 days at 4 °C, and the seedlings were grown on agar plates containing salts and vitamins in half-strength MS medium [43], 2.0% sucrose, and 0.7% agar under short-day conditions (8 h/16 h light/dark cycles) at 22 °C. Ten-day-old seedlings were transferred to soil and grown under long-day conditions (16 h/8 h light/dark cycles) at 22 °C.

### 4.2. Plasmid Construction

To generate an *AtC3H59*-overexpressing construct, the full-length ORF of *AtC3H59* was amplified by PCR and then cloned into pFGL1400, in which the modified CaMV 35S promoter directs the constitutive expression in frame following HA tag [5]. To generate the GUS assay construct, the 242-bp promoter region upstream from the transcription start site of *AtC3H59* was amplified by PCR and cloned into pFGL539 fused to *GUS* [5]. To generate the construct for subcellular localization, the full-length ORF of *AtC3H59* was cloned into a binary vector pFGL1283 in frame with sGFP under the control of a modified CaMV 35S promoter [5].

To generate constructs for analyzing transactivation activity in yeast and yeast two-hybrid assay, full-length ORF and partial fragments of *AtC3H59* were amplified by PCR and cloned into pBD-GAL4 in frame with the GAL4 BD. Full-length ORF and partial fragments of *Desi1* were amplified by PCR and cloned into pGADT7 in frame with GAL4 AD. To generate constructs for the BiFC assay, the full-length ORF of *AtC3H59* and the full-length ORF of *Desi1* were amplified and cloned into the binary vector in frame with an N-terminal YFP and a C-terminal YFP under the control of the CaMV 35S promoter, respectively [44].

The primers used for cloning are listed in Table S3.

### 4.3. Plant Transformation and Selection of Transgenic Plants

The binary vectors were introduced into *Agrobacterium tumefaciens* strain GV3101 (pMP90) using the freeze-thaw method [45]. *Agrobacterium*-mediated Arabidopsis transformation was then performed using the floral-dipping method [46]. Transgenic plants were selected on MS plates containing 50 mg/L kanamycin. Homozygous T_3_ or T_4_ plants were used in this study.

### 4.4. RNA Isolation, cDNA Synthesis, Semi-Quantitative RT-PCR, and Quantitative RT-PCR

Total RNA was isolated using an RNAqueous Kit (Invitrogen, Carlsbad, CA, USA) with Plant RNA Isolation Aid (Invitrogen, Carlsbad, CA, USA), according to the manufacturer’s instructions. Next, 2 μg of total RNA was reverse-transcribed in a total reaction volume of 25 μL; the reaction mixture contained 0.5 μg of oligo-dT primer, 0.5 mM dNTP, 5 μL of 5× reaction buffer, and 200 U of Moloney murine leukemia virus reverse transcriptase (Promega Corp., Madison, WI, USA).

Quantitative RT-PCR was performed in a reaction volume of 20 μL containing 0.4 μL of cDNA, 10 μL of 2× Power SYBR Green PCR Master mix (Applied Biosystems, Foster, CA, USA), and 0.25 μM gene-specific primers. DNA amplification was performed using a QuantStudio^TM^ 3 real-time PCR system (Applied Biosystems, Foster, CA, USA) and analyzed with QuantStudio^TM^ Design & Analysis software (v.1.4.3; Applied Biosystems, Foster, CA, USA). The expression levels of target genes were normalized to the expression levels of *GAPc*. PCR was performed as previously described [47] and all primers used are presented in Table S4.

Semi-quantitative RT-PCR was performed in a reaction volume of 50 μL containing 1 μL of cDNA, 0.5 μM gene-specific primers, 0.5 mM of dNTP, 1 U F-taq DNA polymerase (Solgent, Daejeon, Korea), and 5 μL of 10× reaction buffer. PCR was performed in 32 cycles for *AtC3H59* and 23 cycles for *GAPc*. The number of PCR cycles chosen was within the linear range of the amplification reaction. *GAPc* was amplified as an internal control for the normalization of target gene expression levels. The reaction consisted of an initial denaturation step at 94 °C for 5 min, followed by repeated cycles at 94 °C for 45 s, 56 °C for 45 s, and 72 °C for 45 s, and a final step at 72 °C for 10 min. The primers used for PCR are listed in Table S4.

### 4.5. GUS Activity Analysis

GUS activity was histochemically detected using a protocol described by Seok et al. [5]. Plant tissue was incubated in 2 mM 5-bromo-4-chloro-3-indolyl-β-D-glucuronic acid in 50 mM phosphate buffer (pH 7.0) containing 0.5 mM K_3_Fe(CN)_6_ and 0.5 mM K_4_Fe(CN)_6_ for 6 h at 37 °C. Plant tissue was rinsed with 50 mM phosphate buffer, fixed, and cleared with ethanol (100%):glacial acetic acid (9:1, *v*/*v*) overnight at room temperature.

### 4.6. Protoplast Transformation

*Arabidopsis* protoplast isolation and polyethylene glycol-mediated transformation was performed according to the method described by Yoo et al. [48].

### 4.7. cDNA Library Generation and Yeast Two-hybrid Screening

The Arabidopsis cDNA inserts were introduced into the yeast strain PBN204 with a *Sma*I-linearized pGADT7-Rec vector in three different frames. Each insert DNA was integrated into the pGADT7-Rec vector via yeast homologous recombination.

Yeast two-hybrid screening was performed by PanBioNet (http://www.panbionet.com, accessed on 20 July 2020) using GAL4-BD fused AtC3H59 in pGBKT as bait.

### 4.8. Yeast Transformation and Assay

GAL4 BD-fusion constructs for transactivation activity analysis, or GAL4 BD-fusion constructs and GAL4 AD-fusion constructs for yeast two-hybrid assay were transformed into the yeast strain, YD116, which harbors *GAL1*::*URA3* and *UAS_GAL4_*::*lacZ* as reporter genes. Yeast transformation was performed using the Frozen-EZ Yeast Transformation II^TM^ Kit (Zymo Research Corp., Irvine, CA, USA), according to the manufacturer’s instructions. Transformants were selected on synthetic minimal media lacking tryptophan (SM-Trp) or SM media lacking tryptophan and leucine (SM-Trp/-Leu).

For the yeast growth assay, transformants were streaked onto SM lacking tryptophan and uracil (SM-Trp/-Ura) or SM lacking tryptophan, leucine, and uracil (SM-Trp/-Leu/-Ura) and incubated at 30 °C for 3–5 days. A quantitative β-galactosidase assay using ONPG as a substrate was performed according to the methods described by Miller et al. [49]. The unit of β-galactosidase activity was then calculated using the formula 1000 × OD_420_/(OD_600_ × assay time in min × assay volume in mL). For the β-galactosidase filter assay, the transformants were analyzed using 5-bromo-4-chloro-3-indolyl-β-d-galactopyranoside as a substrate. The β-galactosidase filter assay was performed according to the Clontech Yeast Protocols Handbook (Clontech Laboratories, Inc., Mountain View, CA, USA). The reaction was carried out for 6 h.

### 4.9. Multiple Alignment Analysis

The conserved amino acid sequences were aligned using Clustal Omega (https://www.ebi.ac.uk/Tools/msa/clustalo/, accessed on 20 July 2020) and then manually corrected.

### 4.10. Phylogenetic Tree

A phylogenetic tree was generated using maximum likelihood method in MEGA 7.0.26 software. The number on each node indicates the bootstrap value for 1000 replicates.

## Data Availability

The data presented in this study are available in Supplemental Materials here.

## Supplementary Materials

The following are available online at https://www.mdpi.com/article/10.3390/ijms22094738/s1.
