# Supplementary material for: Non-TZF Protein AtC3H59/ZFWD3 Is Involved in Seed Germination, Seedling Development, and Seed Development, Interacting with PPPDE Family Protein Desi1 in Arabidopsis
Source: Int J Mol Sci. 2021 Apr 29;22(9):4738. doi: 10.3390/ijms22094738 (PMC8124945; doi:10.3390/ijms22094738)
Supplement: Supplementary file 1 [file ijms-22-04738-s001.zip › ijms-1198239-supplementary.pdf]

Supplementary data

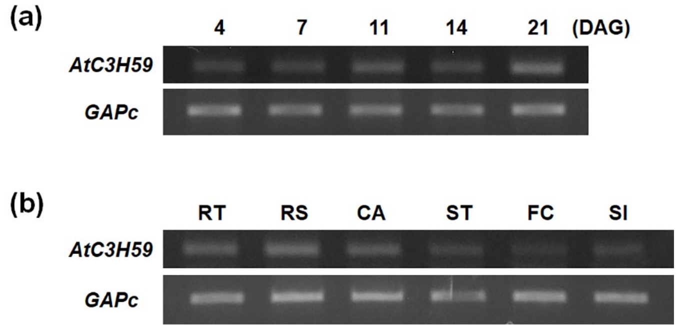

Supplementary Figure S1. Temporal and spatial expression patterns of *AtC3H59*. (**a**) Semi-quantitative RT-PCR analysis of *AtC3H59* in 4-, 7-, 11-, 14-, and 21-day-old WT seedlings grown under SD conditions. (**b**) Semi-quantitative RT-PCR analysis of *AtC3H59* expression in organs of 49-day-old WT grown under LD conditions. RT, roots; RS, rosette leaves; CA, cauline leaves; ST, stems; FC, floral clusters; SI, siliques. *GAPc* was used for an internal control.

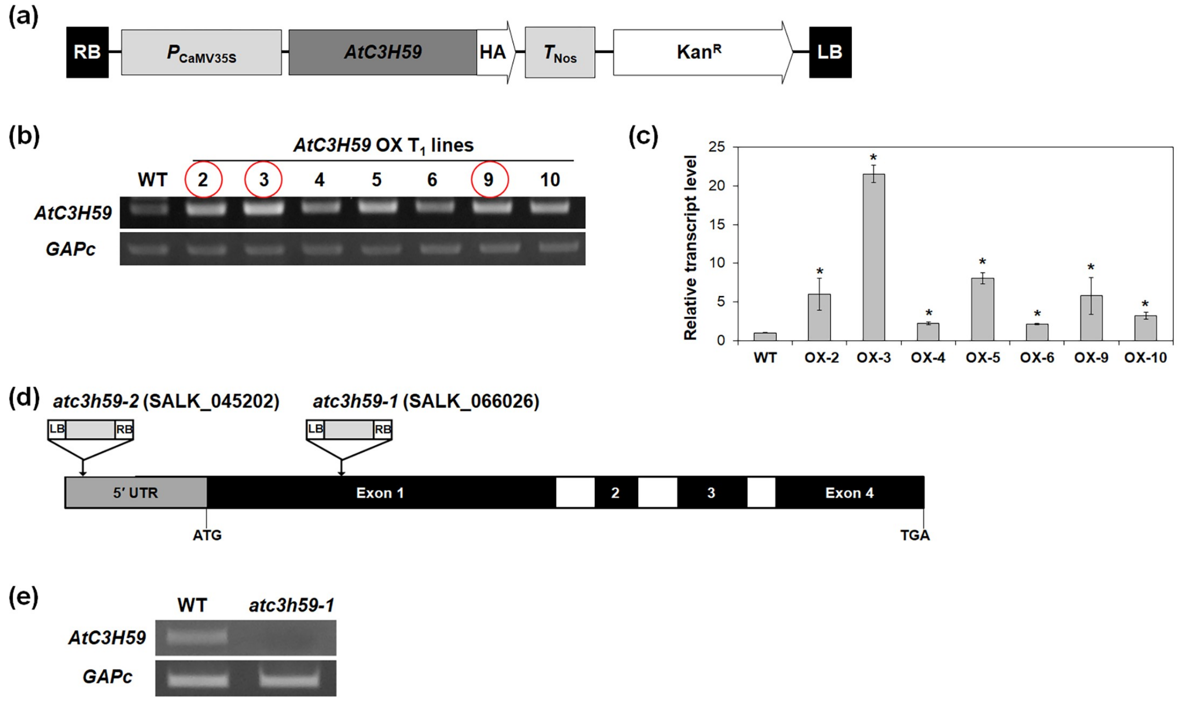

Supplementary Figure S2. Selection of *AtC3H59*-overexpressing transgenic plants (OX) and *atc3h59* mutant. (**a**) Schematic map of vector for overexpression of *AtC3H59*. (**b**) Selection of T_1 lines overexpressing *AtC3H59* by semi-quantitative RT-PCR. Circled lines were selected for further analysis. (**c**) Quantitative RT-PCR analysis of *AtC3H59* in WT and *AtC3H59* OXs. Transcript level in WT was set as 1. Three independent reactions were performed for each technical replicate. Two technical replicates were performed for each biological replicate. Data shown are the mean ± S.D. ($n = 6$). At least two biological replicates showed similar results, with one shown here. * indicate *t*-test $P < 0.05$. (**d**) Genomic structure of *AtC3H59* is shown. Black, gray, and white boxes represent coding sequences, untranslated regions, and intron, respectively. T-DNA insertion sites in *atc3h59-1* (SALK_066026) and *atc3h59-2* (SALK_045202) mutants are depicted by arrow. (**e**) Semi-quantitative RT-PCR analysis of *AtC3H59* transcript levels in 4-week-old WT and *atc3h59-1* mutants. In (**b**), (**c**), and (**e**), *GAPc* was used as an internal control.

Supplementary Table S1. Information of yeast two-hybrid screening. Results of yeast two-hybrid screening using AtC3H59 as a bait and Arabidopsis cDNA library as prey.

**Total no. of transformants**	**No. of positive colonies**	**No. of isolated genes**
5.4×10^6	24 colonies	7 genes

Supplementary Table S2. List of isolated proteins in yeast two-hybrid screening

Locus ID	Gene	No. of selected yeast colonies	Description
At3g07090	Desi1	6	PPPDE putative thiol peptidase family protein
At1g04250	IAA17, AXR3 (Auxin resistant 3)	6	Transcription regulator acting as repressor of auxin-inducible gene expression. Auxin-inducible AUX/IAA gene. Short-lived nuclear protein with four conserved domains.
At5g65670	IAA9 (indole-3-acetic acid inducible 9)	2	Auxin (indole-3-acetic acid) induced gene
At3g13300	VCS (VARICOSE)	4	Encodes VCS (VARICOSE). Involved in mRNA decapping.
At1g71010	FAB1C (Forms aploid and binucleate cells 1C)	2	Encodes a protein that is predicted to act as a phosphatidylinositol-3P 5-kinase, but, because it lacks a FYVE domain, it is unlikely to be efficiently targeted to membranes containing the proposed phosphatidylinositol-3P substrate.
At4g16430	bHLH3, JAM3 (Jasmonate assoicated MYC2 like 3)	3	bHLH3 interacts with JAZ proteins, and functions redundantly with bHLH13, bHLH14, and bHLH17 to negatively regulate jasmonate responses.
At2g45000	NUP62 (Nucleoporin 62), EMB2766 (Embryo defective 2766)	1	Encodes a nucleoporin, a component of the nuclear pore complex, that appears to be a major negative regulator of auxin signaling.

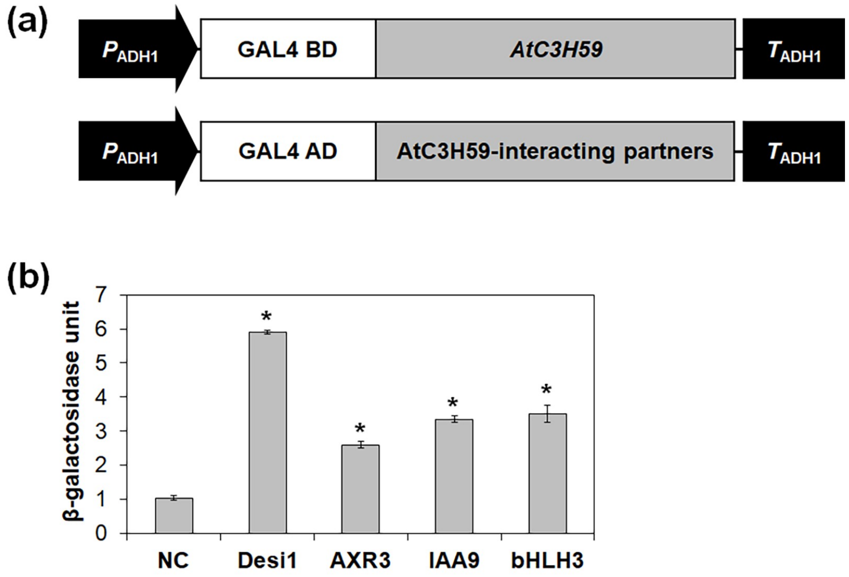

Supplementary Figure S3. Confirmation of interaction between AtC3H59 and its interaction partners in yeast. (**a**) Schematic maps of vectors for yeast two-hybrid of AtC3H59 and its interaction partners. (**b**) Quantitative β-galactosidase ONPG assay. The interaction was quantified by measuring the β-galactosidase activity in yeast extract. Data shown are the means ± S.D. (n = 3). * t-test $P < 0.05$. pBD-GAL4 and pGADT7 were used as a bait and a prey for a negative control, respectively. Locus ID of each gene is as follows: Desi1, At3g07090; AXR3, At1g04250; IAA9, At5g65670; bHLH3, At4g16430. NC, negative control.

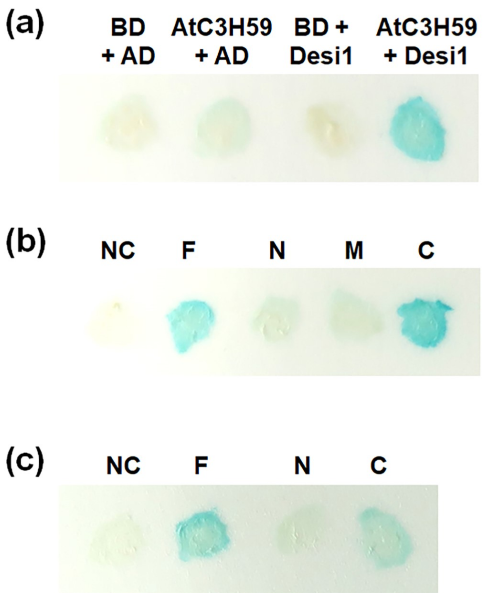

Supplementary Figure S4. Isolation of AtC3H59-interacting protein in yeast. Confirmation of interaction using β-galactosidase filter assay. 5-bromo-4-chloro-3-indolyl-β-d-galactopyranoside was used as a substrate. The reaction was performed for 6 h. pBD-GAL4 and pGADT7 were used as a bait and a prey for a negative control, respectively. In (**b**) and (**c**), NC, negative control; F, full-length ORF; N, N-terminal region; M, middle region; C, C-terminal region.

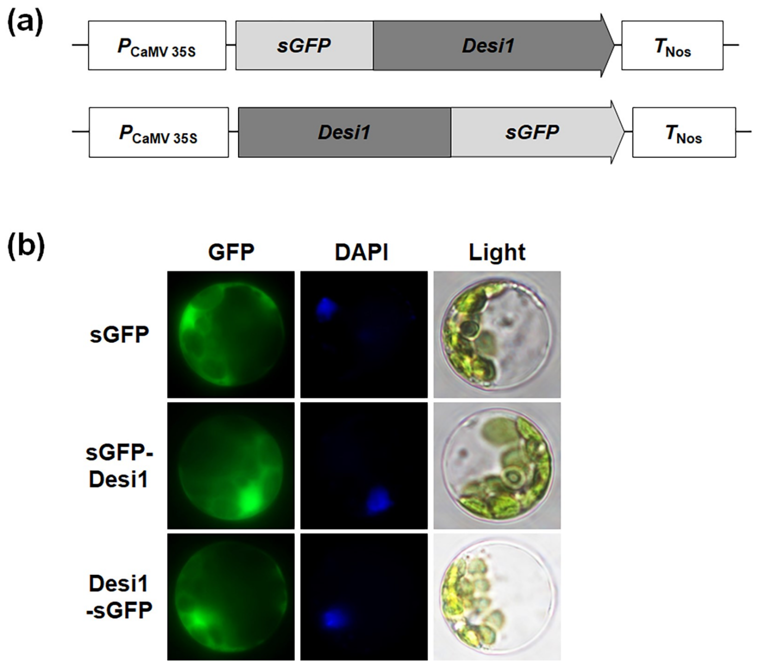

Supplementary Figure S5. Subcellular localization of Desi1. (**a**) Schematic map of sGFP-fused full-length Desi1 constructs. (**b**) Subcellular localization of Desi1 was examined by transient expression of sGFP-Desi1 and Desi1-sGFP fusion proteins in Arabidopsis protoplasts. Left, GFP signal; middle, 4′,6-diamidino-2-phenylindole (DAPI) staining; right, light microscopic picture.

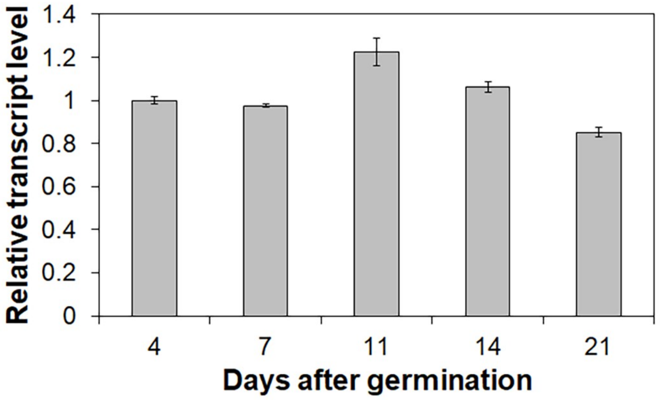

Supplementary Figure S6. Temporal expression patterns of *Desi1*. (**A**) Quantitative RT-PCR analysis of *Desi1* in 4-, 7-, 11-, 14-, and 21-day-old WT seedlings grown under SD conditions. *GAPc* was used for an internal control. Transcript level at 4 DAG was set as 1. Three independent reactions were performed for each technical replicate. Two technical replicates were performed for each biological replicate. Data shown are the mean ± S.D. (*n* = 6). At least two biological replicates showed similar results, with one shown here.

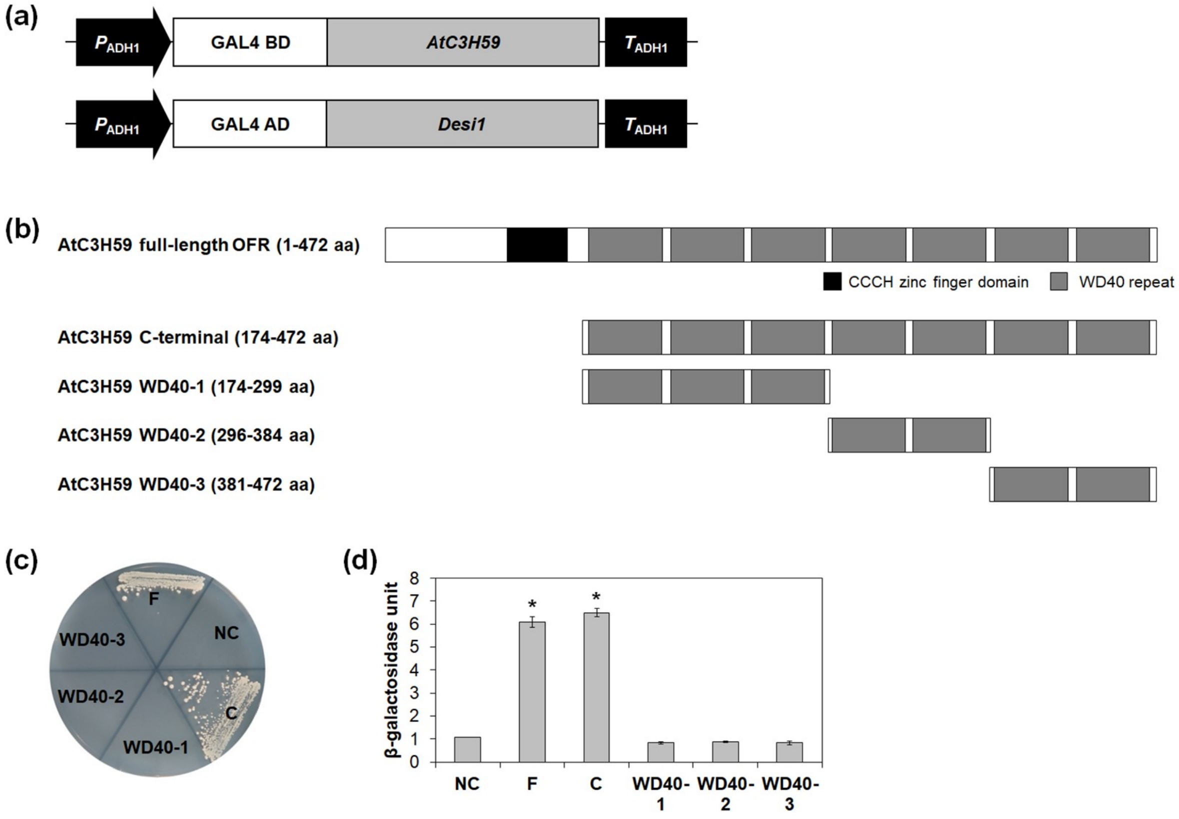

Supplementary Figure S7. Confirmation of protein-interacting domain of AtC3H59. (**a**) Schematic maps of vectors for yeast two-hybrid of AtC3H59 and Desi1. (**b**) Schematic maps of full-length ORF of AtC3H59 and truncated fragments of AtC3H59 for yeast two-hybrid with full-length Desi1. (**c**) Yeast growth assay. Yeast transformants were grown on SM-Trp/-Leu/-Ura. (**d**) Quantitative β-galactosidase ONPG assay. The interaction was quantified by measuring the β-galactosidase activity in yeast extract. Data shown are the means ± S.D. (*n* = 3). * *t*-test $P < 0.05$. In (**c**) and (**d**), pBD-GAL4 and pGADT7 were used as a bait and a prey for a negative control, respectively. NC, negative control; F, full-length ORF; C, C-terminal region.

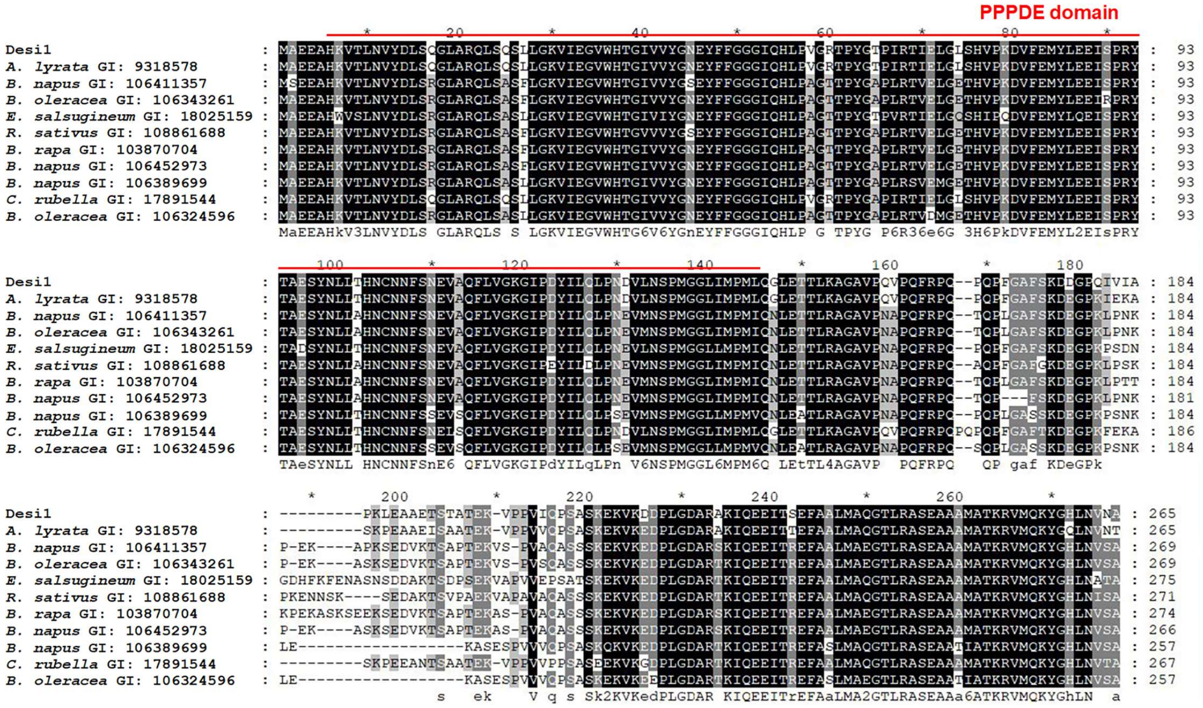

Supplementary Figure S8. Multiple alignment of Desi1 and its orthologs. Multiple sequence alignment was carried out with amino acid sequences of full-length ORF of Desi1 and its orthologs using the Clustal Omega program. One conserved PPPDE domain is annotated.

Supplementary Table S3. List of primers for cloning

Construct	Forward	Reverse
AtC3H59 OX	5′-AGCCTGCAGATGGGT<br>CATCAGTCATCATG-3′	5′-GCTGGATCCAACTTT<br>GGTGCCAGAAGCTA-3′
sGFP-AtC3H59	5′-GGCTCTAGAATGGGT<br>CATCAGTCATCATG-3′	5′-TATCCCGGGCTAAAC<br>TTTGGTGCCAGAAG-3′
$P_{AtC3H59}$::*GUS*	5′-GCGGTCGACTTCCAG<br>TTCTCTCTAATTTT-3′	5′-GCGGGATCCACTGAA<br>ATAAAACTATAGGTC-3′
GAL4 BD-AtC3H59<br>full length ORF	5′-CGCGAATTCATGGGT<br>CATCAGTCATCATG-3′	5′-CGCGTCGACCTAAAC<br>TTTGGTGCCAGAAG-3′
GAL4 BD-AtC3H59<br>N-terminal	5′-CGCGAATTCATGGGT<br>CATCAGTCATCATG-3′	5′-ATAGTCGACGCCATT<br>GAGTCTCGGCCCAC-3′
GAL4 BD-AtC3H59<br>Middle	5′-GCCGAATTCCTCAAT<br>GGCAGTCCCAAAAA-3′	5′-CTAGTCGACTCCAGG<br>AAAACAAGACCAAG-3′
GAL4 BD-AtC3H59<br>C-terminal	5′-TCAGAATTCTTTCCT<br>GGACTGGCCATGGT-3′	5′-CGCGTCGACCTAAAC<br>TTTGGTGCCAGAAG-3′
GAL4 BD-AtC3H59<br>middle + C-terminal	5′-GCCGAATTCCTCAAT<br>GGCAGTCCCAAAAA-3′	5′-CGCGTCGACCTAAAC<br>TTTGGTGCCAGAAG-3′
YFP^{N}-Desi1	5′-ACAGTCGACATGGCT<br>GAGGAAGCGCATAA-3′	5′-CGCGGATCCCTAAGC<br>GTTTACATTGAGAT-3′
YFP^{C}-AtC3H59	5′-CTGGTCGACAGATGG<br>GTCATCAGTCATCATG-3′	5′-GCAGGATCCCTAAAC<br>TTTGGTGCCAGAAG-3′
sGFP-Desi1	5′-GATGTCGACATGGCT<br>GAGGAAGCGCATAA-3′	5′-GCGGGATCCCTAAGC<br>GTTTACATTGAGAT-3′
Desi1-sGFP	5′-GATGTCGACATGGCT<br>GAGGAAGCGCATAA-3′	5′-AATCCCGGGAGCGTT<br>TACATTGAGATGTC-3′
GAL4 AD-Desi1<br>full-length ORF	5′-CGCGAATTCATGGCT<br>GAGGAAGCGCATAA-3′	5′-CGCGGATCCCTAAGC<br>GTTTACATTGAGAT-3′
GAL4 AD-Desi1<br>N-terminal	5′-CGCGAATTCATGGCT<br>GAGGAAGCGCATAA-3′	5′-ACAGGATCCCTATGT<br>GAGGCCTTGCAACA-3′
GAL4 AD-Desi1<br>C-terminal	5′-CGCGAATTCCTCGAA<br>ACAACTCTAAAGGC-3′	5′-CGCGGATCCCTAAGC<br>GTTTACATTGAGAT-3′
GAL4 BD-AtC3H59<br>WD40-1	5′-TCAGAATTCTTTCCT<br>GGACTGGCCATGGT-3′	5′-ACAGTCGACATCAGA<br>CTCCGAGTCTGTAG-3′
GAL4 BD-AtC3H59<br>WD40-2	5′-CGCGAATTCTCGGAG<br>TCTGATCCATTCAA-3′	5′-CGCGTCGACACTTTC<br>ATTTTCAGAACAAG-3′
GAL4 BD-AtC3H59<br>WD40-3	5′-CGCGAATTCGAAAAT<br>GAAAGTTTGAAAGT-3′	5′-CGCGTCGACCTAAAC<br>TTTGGTGCCAGAAG-3′

Supplementary Table S4. List of primers for RT-PCR

Gene	Forward	Reverse	Purpose
GAPc	5′-GTGTCCCAACCGTTGATGTC-3′	5′-TCCCTTGAGTTTGCCTTCGG-3′	Quantitative RT-PCR
AtC3H59	5′- CTTTCAGACACCAGAGGTTG-3′	5′- TTTGGGACTGCCATTGAGTC-3′	
Desi1	5′-TTACCAGTTGGAAGAACTCC-3′	5′- GTGTAACGAGGACTGATTTC-3′	
GAPc	5′-CACTTGAAGGGTGGTGCCAAG-3′	5′-CCTGTTGTCGCCAACGAAGTC-3′	Semi-quantitative RT-PCR
AtC3H59	5′-CTTTCAGACACCAGAGGTTG-3′	5′-TTGCAGCAGTCATTGCATGC-3′	
